# Association between vitamin B6 levels and rheumatoid arthritis: a two-sample Mendelian randomization study

**DOI:** 10.3389/fnut.2024.1442214

**Published:** 2024-10-11

**Authors:** Yanglin Liu, Xukai Wang, Min You, Meiling Zheng, Miao Yu, Xiangyang Leng

**Affiliations:** Changchun University of Chinese Medicine, Changchun, China

**Keywords:** micronutrients, Mendelian randomization (MR), rheumatoid arthritis (RA), immune diseases, causal

## Abstract

**Background:**

Micronutrients play a crucial role in rheumatoid arthritis (RA). Changes in micronutrient levels in RA patients can lead to the worsening of their condition. Though significant correlations between RA and micronutrients have been found in earlier observational studies, their underlying causal relationship is still unknown. This study aimed to elucidate the causal genetic relationships between 15 micronutrients (copper, zinc, magnesium, vitamins A, C, E, D, B6, B12, folate, carotene, iron, selenium, calcium, potassium) and RA.

**Method:**

The exposure factors and outcome data used in the two-sample Mendelian randomization (MR) were derived from publicly available summary statistics data of European populations. The GWAS data for exposure factors were obtained from the OpenGWAS database. For the outcome data of RA, we utilized data from the FinnGen database. We used the MR principle to remove confounding factors and conducted MR analyses using five methods: inverse variance weighted (IVW), MR Egger, weighted median, simple mode, and weighted mode, with IVW as the primary method. Then, we identified micronutrients related to RA and performed MR analyses on these elements, including heterogeneity analysis and pleiotropy analysis such as MR-Egger intercept, MR-PRESSO method, and “leave-one-out” analysis. Finally, we conducted multivariable MR analyses and performed sensitivity analyses again.

**Results:**

The IVW analysis revealed a relationship between vitamin B6 and RA (*p*: 0.029, OR: 1.766, and 95% CI: 1.062–2.938). Sensitivity analysis confirmed the validity and reliability of this result.

**Conclusion:**

This study revealed a causal relationship between vitamin B6 and RA, with vitamin B6 being identified as a risk factor for RA. This finding could contribute to the diagnosis and supplementary treatment of RA patients, providing a reference for subsequent basic research and developing new drugs.

## 1 Introduction

Rheumatoid arthritis (RA) is a complex autoimmune disease (1) that primarily affects the joints, leading to progressive damage. Statistics showed that there were about 460 cases per 100,000 people, with women at higher risk (2). Although the exact cause of RA has remained unclear, research indicated that genetic factors, environmental influences, and abnormal activation of the immune system collectively contribute to the development and progression of the disease (3). RA is characterized by synovial inflammation, typically in symmetrical small joints, causing pain (4). Various cytokines are involved in the immunopathogenesis of RA and play crucial roles. For instance, activated T cells secrete pro-inflammatory factors such as TNF-α and IFN-γ, which trigger joint inflammation and bone destruction, while also activating B cells to produce autoantibodies, thereby worsening RA (5). In the synovium of RA, CD1c^+^ DCs are enriched and secrete chemokines that attract pro-inflammatory immune cells, such as macrophages and monocytes, into the synovium, thereby increasing RA inflammation (6). Interleukins such as IL-6 stimulate RANKL expression in osteoblasts and synovial fibroblasts, promoting osteoclast differentiation and worsening RA (7). Similarly, IL-1β and IL-17 also promote osteoclast formation, aggravating RA through RANKL induction and synergistic interactions with other factors (8). Clinically, RA is incurable, with treatments focusing primarily on symptom relief. JAK inhibitors and comprehensive interventions can effectively alleviate RA symptoms (9). However further research is needed to deepen the understanding of its treatment and prevention (10, 11).

Micronutrients are crucial for maintaining internal balance and health and have a complex and close relationship with immune function. Although the risk factors for RA are not yet fully understood, extensive research has explored their role in RA. Zinc deficiency in RA patients is associated with increased ESR and erosion proteinases, indicating more active disease (12–14). Zinc influx, mediated by Zip8, promotes inflammation in RA patients (15), while zinc supplementation can reduce inflammatory factors, possibly preventing RA development (16). Research indicated that most RA patients have low vitamin D intake (17–20). Vitamin D can improve the severity of RA by decreasing the synthesis of pro-inflammatory mediators (21), reducing NETosis and restoring E-ADA activity in neutrophils (22–25). Studies showed that folic acid and vitamin B12 supplements could reduce cardiovascular risk in RA patients (26). Magnesium deficiency leads to higher levels of TNF and IL-6 in RA, worsening the condition (27). Some micronutrient intakes can exacerbate RA. Despite reduced serum iron levels in RA patients, RA exacerbates local inflammation by decreasing iron in the blood and increasing iron in the synovium (21). Increased inflammation in RA raises serum copper levels, with higher levels correlating with longer disease duration (28, 29). In summary, micronutrients play a crucial role in the pathogenesis of RA, but their relationship with the disease is complex and not clearly defined as positive or negative. Therefore, researching the causal relationship between micronutrients and RA is essential.

MR is considered a “natural experiment,” because it utilizes existing variations in the genetic code to determine exposure to certain conditions and assess whether they affect specific traits (30). MR uses single nucleotide polymorphisms (SNPs) as genetic instruments, providing an alternative method to assess causal evidence between exposure factors and outcome factors. It’s important to note that these genetic variations are randomly allocated, making MR studies less susceptible to reverse causation and confounding factors compared to traditional observational studies (31, 32). MR is based on three main assumptions. First, the genetic variations are associated with exposure. Second, there should be no association between genetic variations and confounding factors. Third, genetic variations only affect the risk of outcome through exposure and not through other pathways. MR is widely used in scientific research and clinical trial exploration due to its advantages in reducing biases and the influence of confounding factors (33). The novelty of this paper lies in the fact that, although RA is associated with various micronutrients, previous studies may be biased due to limited numbers and confounding factors. MR analysis has the potential to explore causal relationships between diseases and characteristics, as it can reduce the effects of confounding factors and reverse causation.

This study aimed to utilize MR analysis to assess the relationships between 15 micronutrients and RA. Specifically, the exposure factors included copper, calcium, iron, magnesium, potassium, selenium, zinc, carotene, folate, vitamin A, vitamin B6, vitamin B12, vitamin C, vitamin D, and vitamin E. The outcome factor was RA. Through this method, the study would explore the potential causal effects of these micronutrients in the pathogenesis of RA, providing new perspectives and potential targets for the prevention and treatment of RA.

## 2 Materials and methods

### 2.1 Study design

**Figure 1:**
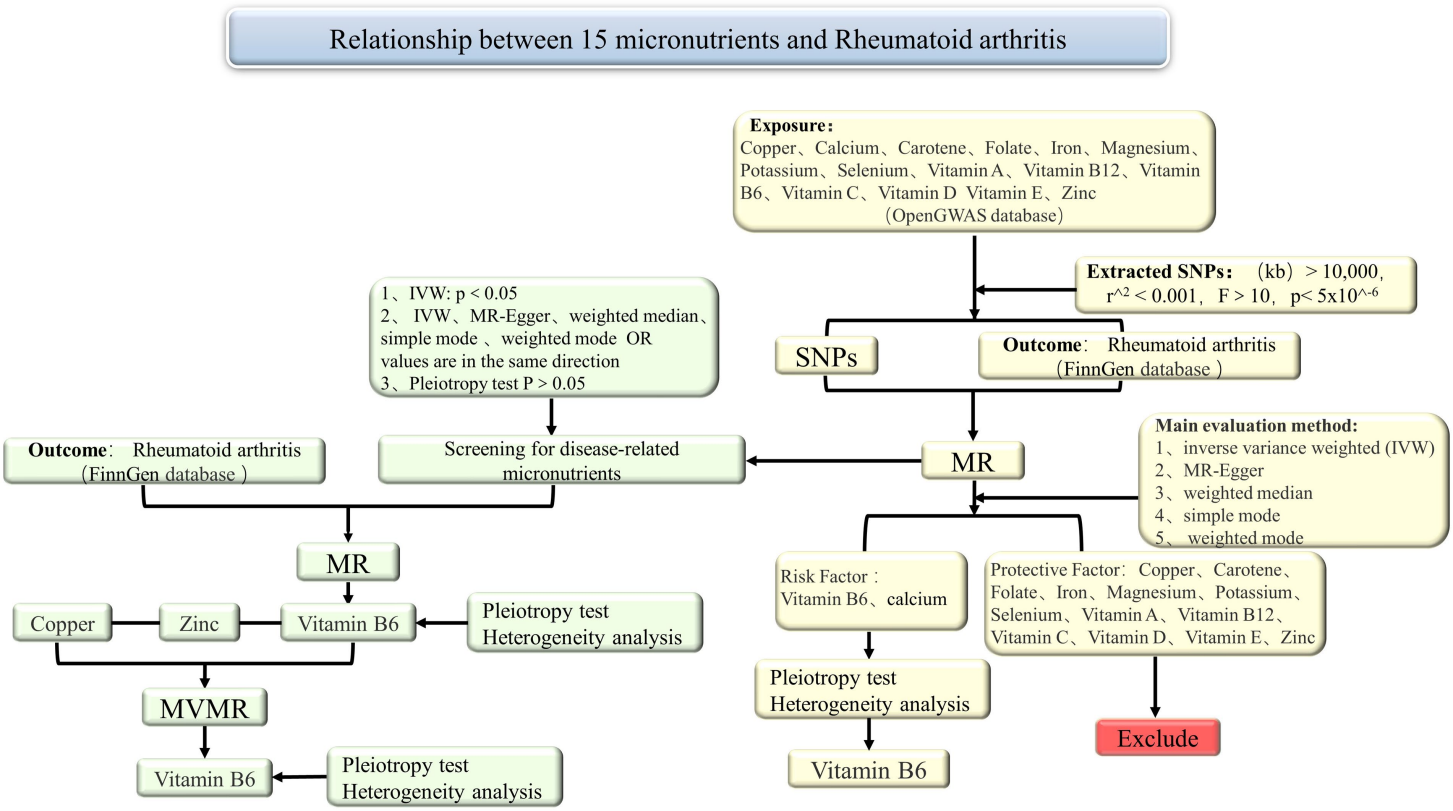
Summary of the MR study design for the relationship between 15 micronutrients and RA. MR, Mendelian randomization; SNPs, single nucleotide polymorphisms; IVW, inverse variance weighted; MVMR, multivariable Mendelian randomization.

The schematic diagram of the study design was shown in Figure 1. In summary, we conducted a two-sample MR study using 15 exposure datasets from publicly available summary statistics in Open GWAS (34), and 1 outcome dataset from publicly available summary statistics in FinnGen. The exposure and outcome data were derived from subjects of European descent to minimize population stratification bias. This MR investigation adhered to three fundamental assumptions. The first assumption was the correlation between instrumental variables (IVs) and exposure. The second assumption was that instrumental variables must be free from any confounding factors related to exposure and outcome. The third assumption was that instrumental variables only affect the outcome through exposure (35, 36).

### 2.2 Data sources

**Figure 2:**
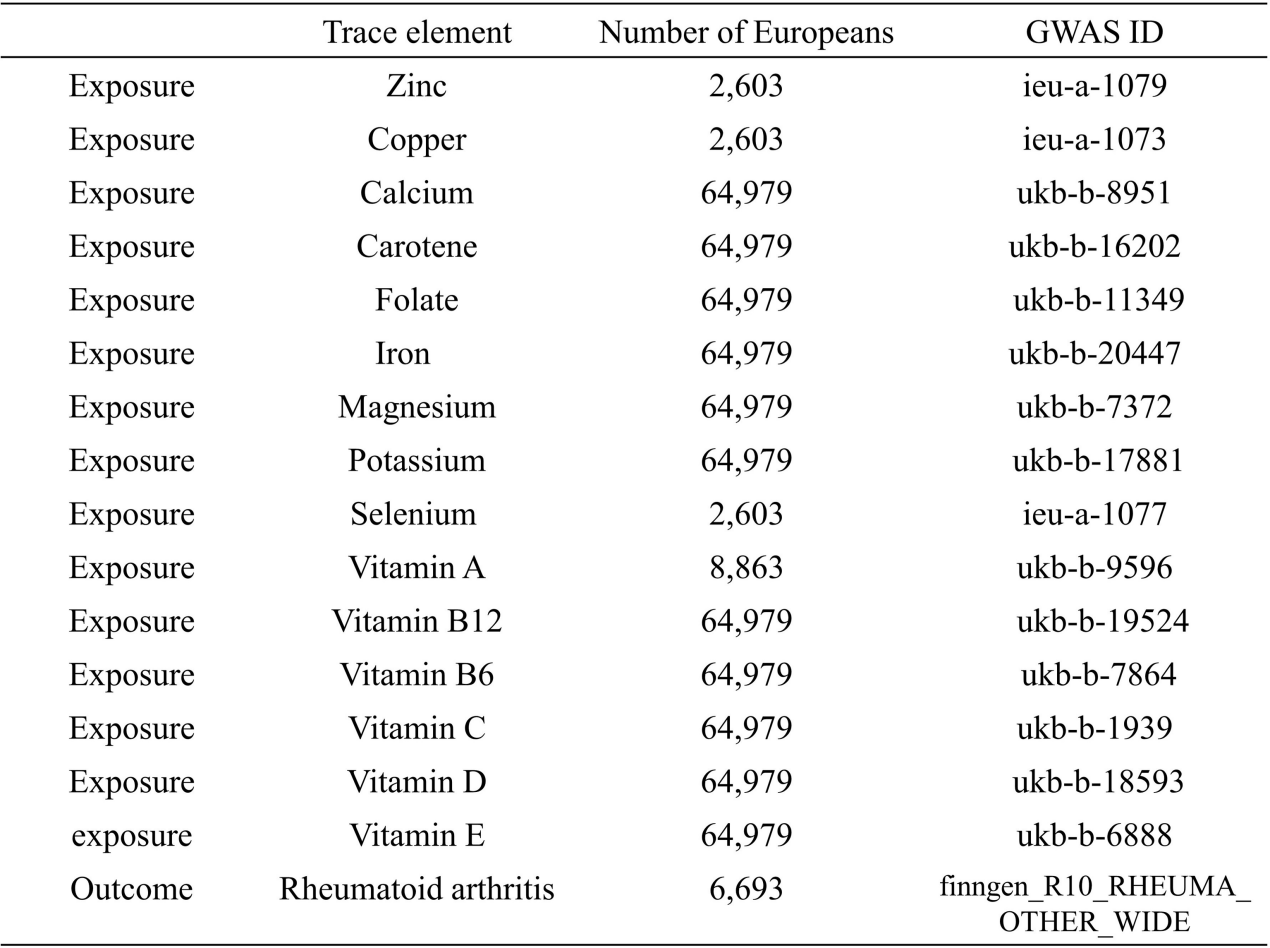
Exposure data from the OpenGWAS database: copper, calcium, iron, magnesium, potassium, selenium, zinc, carotene, folate, vitamins A, B6, B12, C, D, and E. Outcome data from the FinnGen database: rheumatoid arthritis.

This study utilized summary data from genome-wide association studies (GWAS) on copper, calcium, iron, magnesium, potassium, selenium, zinc, carotene, folate, vitamins A, B6, B12, C, D, and E exposures obtained from the OpenGWAS database.^1^ The summary statistics for the 15 micronutrients’ GWAS data were detailed. First, the OpenGWAS data for copper included 2,603 Europeans, with GWAS ID ieu-a-1073. The data for calcium included 64,979 Europeans, with GWAS ID ukb-b-8951. For carotene, the data included 64,979 Europeans, with GWAS ID ukb-b-16202. The data for folate included 64,979 Europeans, with GWAS ID ukb-b-11349. Iron data included 64,979 Europeans, with GWAS ID ukb-b-20447. Magnesium data included 64,979 Europeans, with GWAS ID ukb-b-7372. Potassium data included 64,979 Europeans, with GWAS ID ukb-b-17881. Selenium data included 2,603 Europeans, with GWAS ID ieu-a-1077. Vitamin A data included 8,863 Europeans, with GWAS ID ukb-b-9596. Vitamin B12 data included 64,979 Europeans, with GWAS ID ukb-b-19524. Vitamin B6 data included 64,979 Europeans, with GWAS ID ukb-b-7864. Vitamin C data included 64,979 Europeans, with GWAS ID ukb-b-19390. Vitamin D data included 64,979 Europeans, with GWAS ID ukb-b-18593. Vitamin E data included 64,979 Europeans, with GWAS ID ukb-b-6888. Zinc data included 2,603 Europeans, with GWAS ID ieu-a-1079. Additionally, we obtained outcome data for RA from the FinnGen database.^2^ The RA outcome data (DF10-2023.12.18) under finngen_R10_RHEUMA_OTHER_WIDE included 6,693 RA patients and 405,488 controls. All participants in both exposure and outcome datasets were of European descent (Figure 2).

Detailed information about participants, genotyping, imputation, and quality control can be found on the FinnGen website. Since the data analyzed in this study were all obtained from public databases and were publicly available, institutional review board ethical approval and informed consent were not required. Such data sources ensured transparency and reliability of the data and enabled our research results to be shared and discussed in a wider medical research community.

### 2.3 Selection of instrumental variables

Firstly, to determine the SNPs strongly correlated with exposure in MR analysis, we set the genome-wide significance threshold at 5 × 10^−6^, as instrumental variables (IVs) (37). Subsequently, a linkage disequilibrium region range of >10,000 kb and *r*^2^ < 0.001 was employed to ensure independence among the selected SNPs. Next, we calculated the *F*-statistic for each IV, with the *F*-statistic computed using the following formula: *F* = *R*^2^(*N* − K − 1)/*K*(1 − *R*^2^), to assess the strength of association with the exposure factor, and IVs with *F*-statistics less than 10 were excluded. This approach ensured that we only used SNPs significantly associated with the exposure factor and mutually independent as instrumental variables in the MR analysis, providing robust correlation.

### 2.4 Statistical analysis

In order to assess the potential causal effect of exposure factors on outcome, this study employed the “TwoSampleMR” and “MendelianRandomization” packages in R (version 4.3.3) for two-sample MR analysis, ensuring the reliability and accuracy of the results. Technical assessments were carried out using two-sample MR analysis, including inverse variance weighting (IVW), weighted median, MR-Egger, simple mode, and weighted mode, with IVW as the primary method (38).

The IVW method is a classical MR statistical method that assumes all included SNPs are valid IVs. Each of the five methods has its unique characteristics and assumptions, providing multifaceted causal estimates, followed by heterogeneity and pleiotropy tests. Secondly, exposure factors related to outcome data were screened, and the potential pleiotropy of instrumental variables was evaluated to meet three filtering criteria. Firstly, IVW with *p* < 0.05. Secondly, the consistent direction of OR values among the five methods; and finally, pleiotropy with *p* > 0.05. After screening, MR analysis was conducted on exposure data related to outcome data, using MR Egger’s intercept test and MR-PRESSO to assess horizontal pleiotropy, where *p* > 0.05 indicated no horizontal pleiotropy, followed by heterogeneity testing. Multivariable MR analysis was used for further testing to identify exposure factors that independently produced causal effects on outcome factors. The robustness of MR analysis results was assessed through testing, followed by various sensitivity analyses, including MR-PRESSO testing, pleiotropy analysis, heterogeneity testing, and “leave-one-out” analysis, to examine whether individual SNP affected the relationship assessment between exposure and outcome.

## 3 Results

### 3.1 MR analysis

**Figure 3:**
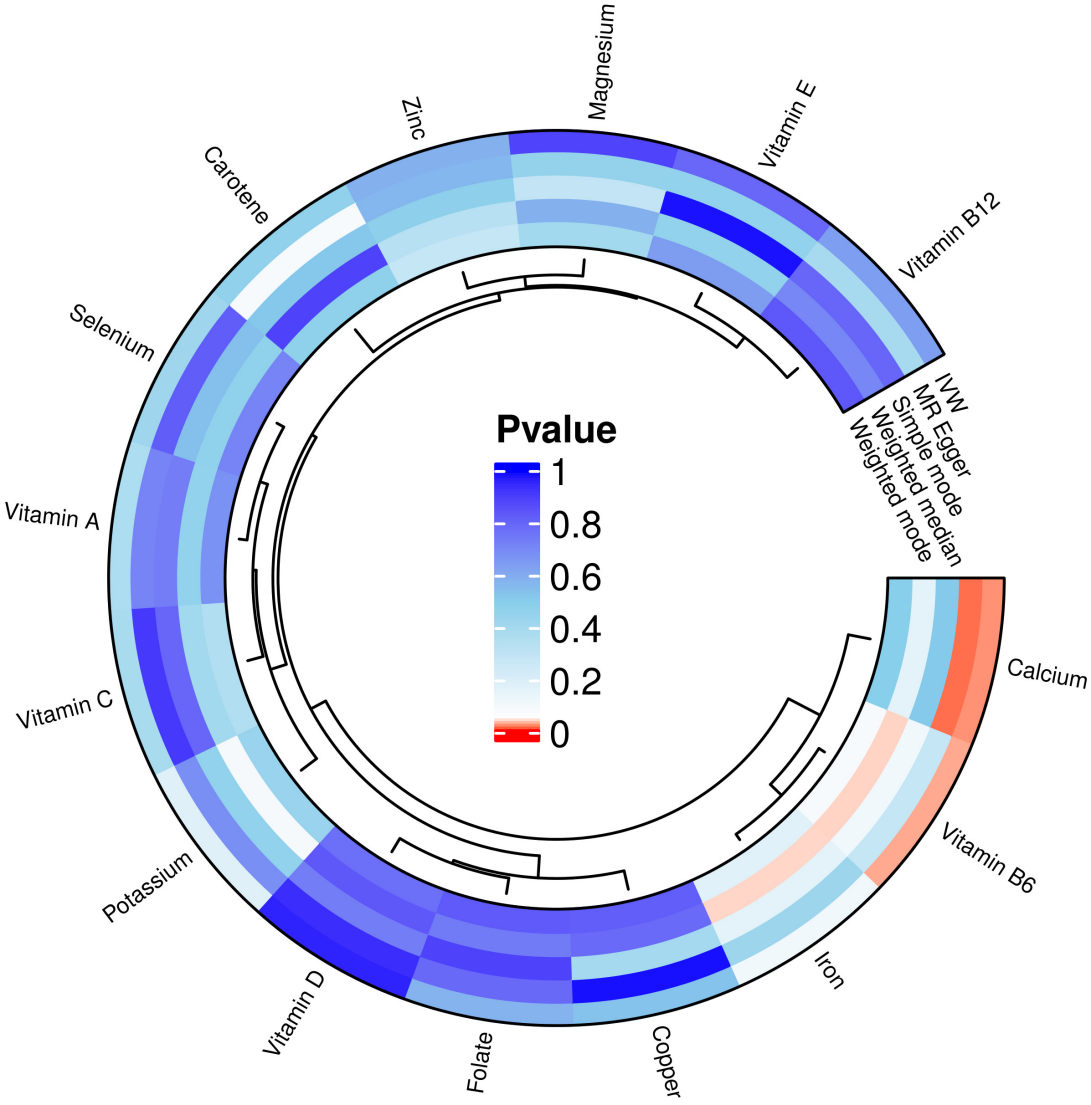
MR analysis results of exposures (copper, calcium, iron, magnesium, potassium, selenium, zinc, carotene, folate, vitamins A, B6, B12, C, D, and E) and outcome (RA). Five methods: inverse variance weighting (IVW), weighted median, MR-Egger, simple mode, and weighted mode.

Copper, calcium, iron, magnesium, potassium, selenium, zinc, carotene, folate, vitamin A, vitamin B6, vitamin B12, vitamin C, and vitamin D, vitamin E, these 15 micronutrients were considered as exposure factors, and RA was considered as the outcome factor, for MR analysis (Supplementary File S1) and visualized the results (Figure 3). After multiple tests and corrections, the random-effects IVW analysis indicated that calcium (*p*: 0.022, OR: 0.683, and 95% CI: 0.493–0.948) and vitamin B6 (*p*: 0.028, OR: 1.427 95% CI: 1.039–1.959) have a significant causal relationship with RA. Vitamin B6 is a risk factor for RA. Carotene, selenium, vitamins A, C, D, E, B12, iron, copper, zinc, magnesium, folate and potassium with IVW *p* > 0.05 were excluded. In addition, the results obtained from the MR-Egger analysis for calcium were consistent with IVW, but the results obtained from the weighted median, simple mode, and weighted mode analyses were opposite to IVW results, without heterogeneity (*p* > 0.05), with OR values <1. The results obtained from the weighted median analysis for vitamin B6 were consistent with IVW, but the results obtained from MR-Egger, simple mode, and weighted mode analyses showed no heterogeneity (*p* > 0.05), with OR values >1.

**Figure 4:**
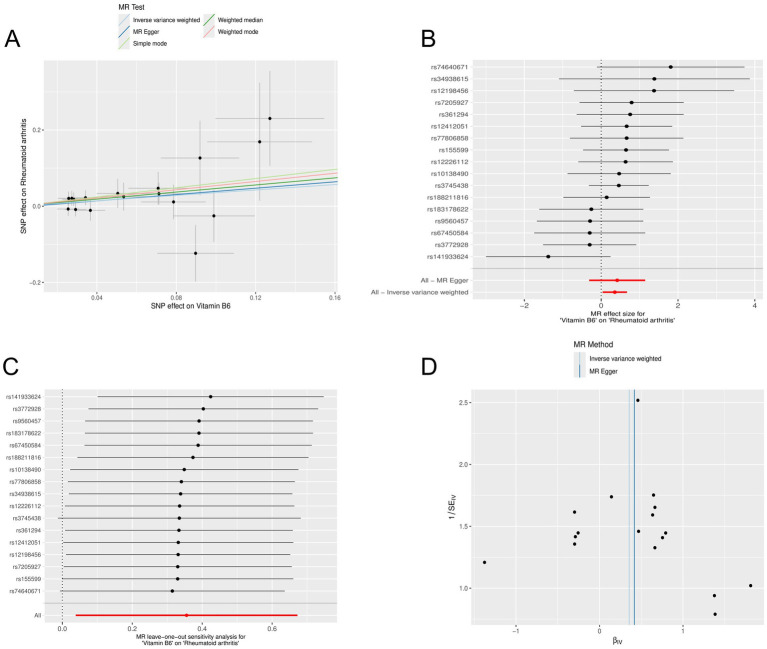
Mendelian randomization analysis of the causal relationship between vitamin B6 and rheumatoid arthritis. (A) Scatterplots for the causal association between vitamin B6 and rheumatoid arthritis. The slope of a straight line indicates the magnitude of causality. Black dots represent genetic instruments included in the main Mendelian randomization analysis. (B) Forest map visualization of the causal impact of each SNP on rheumatoid arthritis risk. (C) “Leave-one-out” plots for the causal association between vitamin B6 on rheumatoid arthritis risk. (D) Funnel plot showing heterogeneity of SNP.

We used the R software package to screen for micronutrients related to the disease. After analysis, it was found that the multifunctionality analysis of calcium had a *p*-value of 0.044 (*p* < 0.05), indicating that it influenced the outcome through factors other than the exposure factor, indicating pleiotropy. Therefore, it could not be considered a valid result in the study and was excluded. However, the multifunctionality of vitamin B6 had a *p*-value of 0.857 (*p* > 0.05), which was related to RA (Supplementary File S2). MR analysis was conducted on vitamin B6 related to RA (Supplementary File S3). Random-effects IVW analysis showed that vitamin B6 (*p*: 0.028, OR: 1.427, and 95% CI: 1.039–1.959), and conducted pleiotropy testing (Supplementary File S4) and heterogeneity testing (Supplementary File S5), both showing *p*-values greater than 0. The results of outlier detection indicated that the comprehensive *p*-value of all outlier detections was >0.05 (Supplementary File S6), and there were no SNPs in the outlier detection of individual SNP (Supplementary File S7). Additionally, we observed through scatter plots that the results of SNPs on exposure and outcome factors were consistent across the five methods. Forest plots generated using MR Egger and IVW methods showed that SNPs effect sizes were greater than 0. “Leave-one-out” sensitivity analysis indicated that removing a single SNP did not excessively influence the MR analysis. The funnel plot also displayed a symmetrical distribution (Figure 4).

### 3.2 Multivariable MR analysis

**Figure 5:**
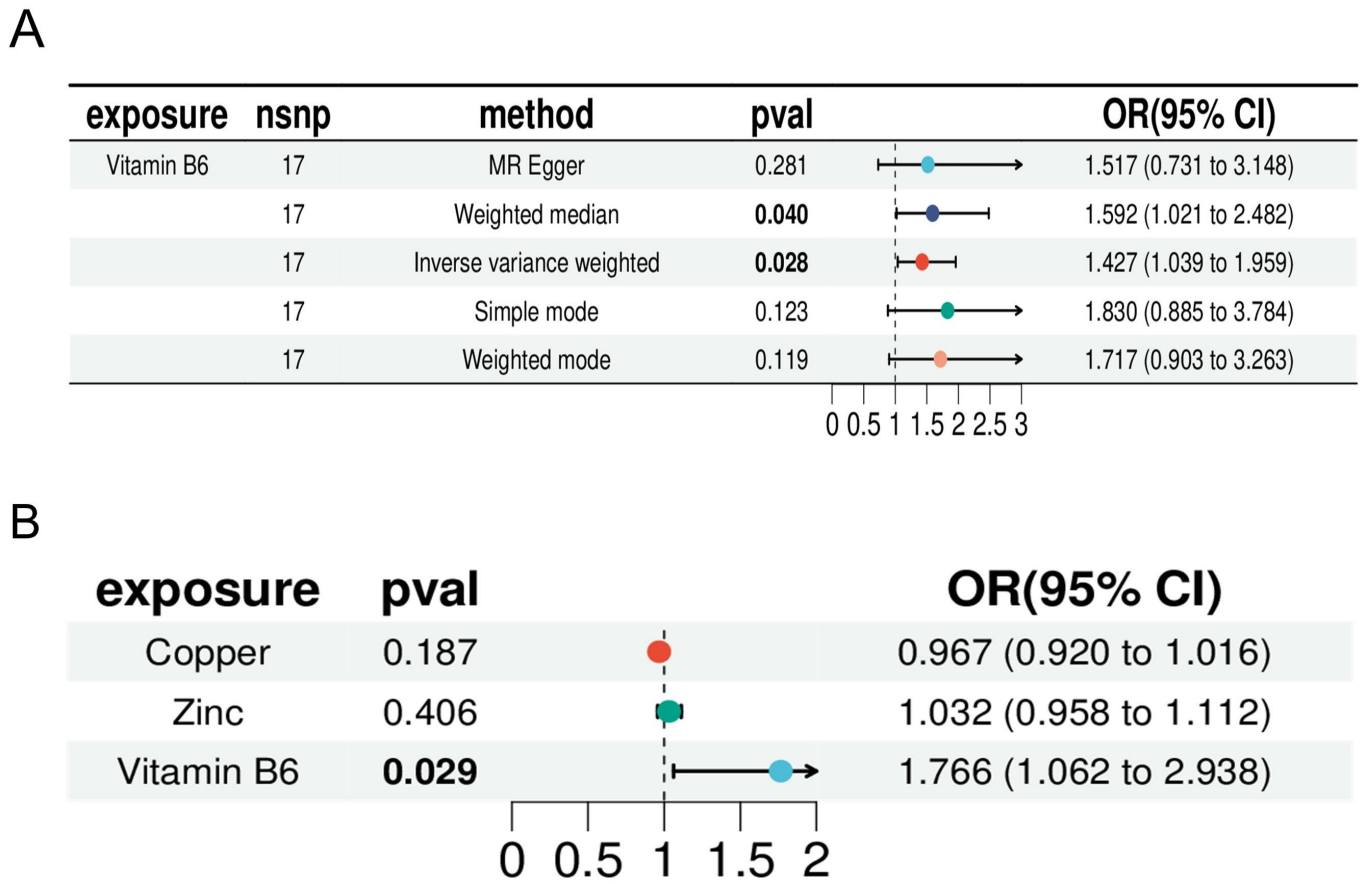
Forest plot. (A) Forest plot of Mendelian randomization analysis of vitamin B6 with inverse variance weighting (IVW), weighted median, MR-Egger, simple mode, and weighted mode. (B) Forest plots for inverse variance-weighted Mendelian randomization analysis of zinc, copper, and vitamin B6.

We selected zinc and copper, two micronutrients related to vitamin B among the 15 micronutrients, for multivariable MR (Supplementary File S8). The results indicated that zinc (*p*: 0.406, OR: 1.032, and 95% CI: 0.958–1.112) and copper (*p*: 0.967, OR: 1.032 95% CI: 0.920–1.016) did not have independent causal effects on RA and were therefore excluded. Vitamin B6 (*p*: 0.029, OR: 1.766, and 95% CI: 1.062–2.938) had an independent causal effect on RA, indicating that vitamin B6 is a risk factor for RA. Heterogeneity and multifunctionality tests were conducted, obtaining *Q* values with *p* > 0.05, indicating no significant heterogeneity or multifunctionality in the data (Supplementary File S9). We compared the forest plot of vitamin B6 with the combined forest plot of zinc, copper, and vitamin B6, which demonstrated the causal relationship between vitamin B6 and RA (Figure 5). Therefore, we identified vitamin B6 as a potential risk factor for RA.

## 4 Discussion

RA is a complex autoimmune disease with an incompletely understood pathophysiology. RA patients often experience decreased bone density, bone loss, and osteoporosis due to inflammation, leading to bone destruction closely related to micronutrient levels (39, 40). This study employed MR to investigate the causal relationships between 15 micronutrients (copper, calcium, iron, magnesium, potassium, selenium, zinc, carotene, folate, vitamin A, vitamin B6, vitamin B12, vitamin C, vitamin D, and vitamin E) and RA. The analysis revealed a significant causal link between RA and vitamin B6, suggesting that vitamin B6 is a risk factor for RA.

Vitamin B6 is essential for maintaining normal metabolism and immune responses, and it exhibits anti-inflammatory properties. It is involved in generating auxiliary factors in metabolic pathways, promoting an immune response (41). Research has also indicated that vitamin B6 reduced the expression of pro-inflammatory cytokines by inhibiting the NF-κB and mitogen-activated protein kinase signaling pathways. Moreover, it could reduce the accumulation of sphingosine-1-phosphate through a sphingosine-1-phosphate lyase-dependent mechanism, thereby preventing excessive inflammation accumulation (42). Vitamin B6 also plays a crucial role in the production of T lymphocytes and cytokines. Therefore, a deficiency in vitamin B6 can affect humoral and cell-mediated immune responses, as well as the differentiation and maturation of lymphocytes (43), leading to decreased immunity, including reduced formation of serum antibodies, decreased production of IL-2, and increased production of IL-4. Under conditions of chronic inflammation, vitamin B6 is negatively correlated with levels of IL-6 and TNF-α (34).

The intake of vitamin B6 has been linked to RA progression. According to the Food and Nutrition Board of the Institute of Medicine in the United States, the recommended dietary allowance (RDA) for vitamin B6 ranges from 1.3 mg (young people) to 1.7 mg (adult males) (32), and up to 2 mg for lactating women (44). Consuming at least 5 mg/day of vitamin B6 helps prevent significant increases in inflammation (45). Among 24,151 RA patients, researchers found a negative correlation between vitamin B6 intake and RA risk (*p* < 0.001) (37). Research has indicated that a high daily intake of vitamin B6 supplements (100 mg/day) could suppress the levels of pro-inflammatory cytokines, specifically IL-6 and TNF-α, in patients suffering from RA (46). At the same time, the plasma pyridoxal-phosphate (PLP) in RA patients could reflect their functional vitamin B6 status (47). These studies indicated that vitamin B6 has a protective effect against RA. The severity of RA was associated with vitamin B6 biomarkers, and the relationship between vitamin B6 and disease activity in RA patients could be observed before and after TNF-α inhibitor treatment (48).

However, our MR study presented a new perspective, suggesting that vitamin B6 may be a potential risk factor for RA, rather than the protective factor previously believed. This discrepancy may stem from the complexity and context-dependency of vitamin B6’s role in immune regulation and inflammatory responses. While previous research has shown that vitamin B6 can reduce the expression of pro-inflammatory cytokines such as IL-6 and TNF-α, its broader functions within the metabolic and immune systems may vary based on disease stage, genetic susceptibility, or environmental factors. Nevertheless, our MR analysis, using genetic variation as an instrumental variable, reveals a different scenario—elevated levels of vitamin B6 may be involved in the pathogenesis of RA, potentially exacerbating rather than alleviating autoimmune responses through unclear immune-regulatory pathways. This contrast in findings raised important questions about the dose-response relationship between vitamin B6 and RA, and it may have widespread implications for personalized medicine in managing chronic inflammatory diseases. The dual nature of vitamin B6—acting as both an anti-inflammatory agent and a potential risk factor—indicated that supplementation strategies should be carefully adjusted for individuals at risk for RA. Future research should focus on the precise role of vitamin B6 at different stages of RA progression and its interactions with other micronutrients and genetic factors. These findings highlighted the need for a cautious interpretation of vitamin B6’s role in RA and advocate for a balanced and personalized approach to its supplementation and therapeutic use in autoimmune disease management.

Our study provided new insights into the pathogenesis of RA, but it had to be acknowledged that our study had limitations. To reduce the risk of population stratification, the data we stratified only by European ancestry, without considering factors such as age, diet, and micronutrient intake, which might have introduced bias into the results. Additionally, with the genome-wide significance level set at 5 × 10^−8^, we did not have a sufficient number of SNPs for MR analysis. Therefore, we expanded the genome-wide significance level to 5 × 10^−6^, although this adjustment was reasonable, it still had limitations. Although our MR study suggested that vitamin B6 is a potential risk factor for RA, previous studies have often highlighted the protective effects of vitamin B6, such as its ability to reduce inflammation and modulate immune responses. The differences in findings may be due to variations in study design, population characteristics, and vitamin B6 intake levels. Future research should further explore the specific mechanisms by which vitamin B6 operates in different stages of RA progression, while taking into account factors such as genetic background, dietary habits, and micronutrient intake. This will help to better understand the dual role of vitamin B6 in RA and its complexity in immune regulation and inflammation response.

## 5 Conclusion

In conclusion, our findings indicated that vitamin B6 is a potential risk factor for RA. However, our research did not establish a causal relationship between other micronutrients and RA. We hope that future researchers will employ methods such as randomized controlled trials and mechanistic studies to further explore the safety, toxicity, and dosage of vitamin B6, investigate its relationship with RA and potential mechanisms, and thereby provide more reliable evidence for future studies.

## Data Availability

The original contributions presented in the study are included in the article/Supplementary material, further inquiries can be directed to the corresponding author.

## Footnotes

^1^https://gwas.mrcieu.ac.uk/

^2^https://www.finngen.fi/

## Supplementary material

SUPPLEMENTARY FILE S1 Results of Mendelian randomization of 15 exposure and outcome factors.

SUPPLEMENTARY FILE S2 Screening for disease-associated micronutrients Mendelian randomization of polytropic results.

SUPPLEMENTARY FILE S3 MR analysis of the micronutrient vitamin B6 associated with RA.

SUPPLEMENTARY FILE S4 Results of pleiotropy analysis.

SUPPLEMENTARY FILE S5 Results of heterogeneity analysis.

SUPPLEMENTARY FILE S6 Results for all outliers detected.

SUPPLEMENTARY FILE S7 Results of testing for individual SNP outliers.

SUPPLEMENTARY FILE S8 Results of multivariate Mendelian randomization for vitamin B, zinc, and copper.

SUPPLEMENTARY FILE S9 Results of heterogeneity and multifunctionality tests.
